# Safety confirmation of induced pluripotent stem cell-derived cardiomyocyte patch transplantation for ischemic cardiomyopathy: first three case reports

**DOI:** 10.3389/fcvm.2023.1182209

**Published:** 2023-09-15

**Authors:** Takuji Kawamura, Yoshito Ito, Emiko Ito, Maki Takeda, Tsubasa Mikami, Takura Taguchi, Noriko Mochizuki-Oda, Masao Sasai, Tomomi Shimamoto, Yukako Nitta, Daisuke Yoshioka, Masashi Kawamura, Ai Kawamura, Yusuke Misumi, Yasushi Sakata, Yoshiki Sawa, Shigeru Miyagawa

**Affiliations:** ^1^ Department of Cardiovascular Surgery, Osaka University Graduate School of Medicine, Suita, Japan; ^2^ Department of Cardiology, Osaka University Graduate School of Medicine, Suita, Japan; ^3^ Devision of Health Sciences, Osaka University Graduate School of Medicine, Suita, Japan

**Keywords:** cardiomyocyte patch transplantation, iPS cells, myocardial regenerative medicine, heart failure, immune response, HLA antibodies

## Abstract

**Introduction:**

With the expected increase in patients with heart failure and ischemic 15 cardiomyopathy, the development of myocardial regenerative medicine using cell transplantation as a novel treatment method is progressing. This first-in-human clinical trial aimed to confirm the safety of cardiomyocyte patch transplantation derived from allogeneic induced pluripotent stem (iPS) cells based on the results of several preclinical studies.

**Study design:**

The inclusion criteria were left ventricular ejection fraction of 35% or less; heart failure symptoms of New York Heart Association class III or higher despite existing therapies such as revascularization; and a 1-year observation period that included a 3-month immunosuppressive drug administration period after transplantation of iPS cell-derived cardiomyocyte patches to evaluate adverse events, cardiac function, myocardial blood flow, heart failure symptoms, and immune response.

**Results:**

In the first three cases of this trial, no transplanted cell-related adverse events were observed during the 1-year observation period, and improvement in heart failure symptoms was observed. In addition, improvements in left ventricular contractility and myocardial blood flow were observed in two of the three patients. Regarding immune response, an increase in transplant cell-specific antibody titer was observed in all three patients after immunosuppressive drug administration. In one patient with poor improvement in cardiac function and myocardial blood flow, an increase in antibody titer against HLA-DQ was observed even before cell transplantation.

**Conclusions:**

Our case findings demonstrate that the transplantation of iPS cell-derived cardiomyocyte patches for ischemic cardiomyopathy can be safely performed; however, further investigation of the therapeutic effect and its relationship with an immune response is needed by accumulating the number of patients through continued clinical trials.

## 1. Introduction

The number of patients with ischemic cardiomyopathy is increasing worldwide with the aging population (1). Ischemic cardiomyopathy generally worsens from the onset of heart failure symptoms to repeated remission and exacerbation. Left ventricular assist devices and heart transplants are indicated for patients who continue to deteriorate despite receiving appropriate medical therapy, coronary revascularization, and structural interventions for valvular disease (2). However, these replacement therapies have complications and lack donors; therefore, the indications for such treatments have to be limited. In this context, we previously conducted basic research and preclinical studies on induced pluripotent stem cell (iPSC)-derived cardiomyocyte (iPSC-CM) transplantation as an angiogenesis therapy by cell patch transplantation and demonstrated that it is promising in terms of safety and therapeutic efficacy (3–5). Therefore, as previously reported in a study (6), we started an investigator-initiated clinical trial of iPSC-CM patch transplantation as a new treatment method for ischemic cardiomyopathy. The primary objective of this clinical trial was to confirm the safety of iPSC-CM transplantation in a clinical setting. Potential adverse events include undifferentiated cell-derived tumor formation that may persist in transplanted iPSC-CMs and immune reactions to allografted cells. Based on previous preclinical studies, HLA matching is not required for immunosuppressive therapy for allogeneic iPSC-CM transplantation. Therapeutic effects can be obtained by performing immunosuppressive treatment similar to normal heart transplantation (7). However, the immune response that occurs in actual clinical situations remains unknown. Therefore, we review and report about three cases, including those already reported, who underwent iPSC-CM patch transplantation, and present data on general clinical outcomes, safety assessment, and immune reactions.

## 2. Materials and methods

### 2.1. Ethical committee approval

This clinical trial was approved by the ethical review committee of Osaka University (#199006-A), the Ministry of Health, Labor, and Welfare, Japan (#2019-143), and registered in the Japan Registry of Clinical Trials (https://jrct.niph.go.jp/en-latest-detail/jRCT2053190081). Written informed consent was obtained from all individuals and was saved according to the clinical trial plan.

### 2.2. Study design

Patients who met the inclusion criteria mentioned below and confirmed that they did not meet any exclusion criteria were included in the study after obtaining written informed consent. After iPSC-CM patch transplantation, immunosuppressants, including tacrolimus, mycophenolate mofetil, and prednisolone, were administered for 3 months, including the tapering period. Data on adverse events 1 year after transplantation were collected to determine the causal relationship with the iPSC-CM patch. Serial cardiac function was evaluated using echocardiography and cardiac computed tomography (CT), and myocardial blood flow was measured using NH_3_-positron emission tomography (PET). In addition, the immune response to transplanted cells was evaluated by quantifying the antibodies against the HLA of the cells in the serum.

As this investigator-initiated clinical trial will be the first-in-human trial using iPSC-CMs, this study mainly evaluated the safety of the treatment. To avoid potential bias, the decision to continue this trial was left to an external evaluation committee after the first three cases.

### 2.3. Inclusion criteria

The inclusion criteria were a diagnosis of ischemic cardiomyopathy with a left ventricular ejection fraction (LVEF) of 35% or less and heart failure symptoms of New York Heart Association class III and IV after standard treatment, including coronary revascularization and medication therapy.

### 2.4. Exclusion criteria

The exclusion criteria were as follows: (1) autoimmune disease, (2) allergy or hypersensitivity to the immunosuppressant, (3) severe infection, (4) persistent shock due to worsening heart failure, (5) irreversible organ failure other than heart failure, (6) malignancy, (7) pregnancy, (8) alcoholic or drug addiction in recent 6 months, (9) allergies or hypersensitivity to animals used to obtain raw materials, and (10) severe pulmonary hypertension.

### 2.5. Operative procedure

As previously reported (6), three iPSC-CM patches, with 3.3 × 107 cells per patch, the form of which was circular about 3.5 cm in diameter, were transplanted on the left ventricular anterior and the lateral wall under general anesthesia (Supplementary Figure S1). The pericardium was incised through the left fourth or fifth intercostal thoracotomy to observe the surface of the myocardium. Then the patches were fixed to the lateral side of the left anterior descending artery to cover the anterior to the lateral wall of the left ventricle with 7-0 prolene thread, if necessary, with fibrin glue spread on the patches to prevent shifting. The procedure is then terminated with sparse closure of the pericardium. As for the location of the cell patch, we observe the condition of the cardiac surface and choose a location where myocardial viability could be expected. If needed, intra-aortic balloon pumping (IABP) was also performed during surgery.

### 2.6. Anti-HLA antibody evaluation

Postoperative serum samples from each patient were examined over time for the presence or absence of anti-HLA antibodies that are specific or cross-reactive to the transplanted cells to evaluate the immune response against transplanted iPSC-CMs. LABScreen™ Single Antigen (One Lambda, Inc., Los Angeles, California, United States) was used in outsourced inspections to HLA laboratories (https://hla.or.jp) to identify or quantify anti-HLA antibodies, and the immune response was quantified using fluorescence intensity of conjugated antibodies against anti-HLA antibodies.

## 3. Results

### 3.1. Preoperative status and indication for the clinical trial

#### 3.1.1. Case 1

As previously reported (6), a 51-year-old man who temporarily became dependent on mechanical circulatory support (MCS) after two episodes of acute myocardial infarction was weaned off MCS. Cardioprotective drugs, including beta-blockers and ACE inhibitors, were administered, and despite the maximum possible medical treatment, symptoms of heart failure of NYHA class III persisted. Echocardiography revealed a low LVEF of 30%, meeting the inclusion criteria for this study and no applicable exclusion criteria.

#### 3.1.2. Case 2

A 76-year-old man developed an inferior wall infarction 18 years ago and had stents placed in the right coronary artery (RCA) #1 and #3 and left circumflex artery (LCx) #13. He developed an anterior wall infarction 13 years ago, and a stent was placed in the left anterior descending artery (LAD) #7; however, unstable angina pectoris developed 1 year later, and a drug-eluting stent (DES) was placed in LAD #6. Finally, DES was placed in the LCx 7 years ago, and since then, follow-up at another hospital has been continued. At the time of referral to our hospital, there was significant stenosis of the RCA and LCx, and percutaneous coronary intervention (PCI) was performed. Besides technically possible PCI, beta-blockers and mineralocorticoid receptor antagonists were administered as cardioprotective drugs, although the heart failure symptoms of NYHA class III persisted. Echocardiography at our hospital revealed an LVEF of 21%, which met the inclusion criteria for this trial. The preoperative screening analysis did not reveal any findings that met the exclusion criteria.

#### 3.1.3. Case 3

A 65-year-old man underwent CABG and MVP for acute myocardial infarction and papillary muscle rupture 5 years ago. Oral beta-blockers and angiotensin receptor blockers were administered as cardioprotective drugs. The patient had heart failure symptoms of NYHA class III and a lower LVEF of 35%, despite angiographic patent bypass grafts, which met the inclusion criteria for this study and did not meet the relevant exclusion criteria (Table 1).

**Table 1:** Preoperative findings.

	Age (years)	Sex	BH (cm)	BW (kg)	Coronary revascularization	Medication	Heart failure symptom
Case 1	51	Male	167.4	58.8	PCI	Carvedilol Candesartan Spironolactone	NYHA class III
Case 2	76	Male	166.2	64	PCI	Carvedilol Spironolactone	NYHA class III
Case 3	65	Male	159.7	67.8	CABG	Carvedilol Telmisartan	NYHA class III

BH, body height; BW, body weight; PCI, percutaneous coronary intervention; CABG, coronary artery bypass grafting; NYHA, New York Heart Association.

### 3.2. Operation

In cases 1 and 2, IABP was used to support cardiac function. Because case 3 was a prior CABG case, iPSC-CM transplantation was performed after detaching the adhesions in the pericardium. The operation times were 116, 97, and 125 min in cases 1, 2, and 3, respectively. All three patients returned to the ICU while intubated, IABP was removed, and patients were extubated while monitoring hemodynamics.

### 3.3. Severe adverse events

Four severe adverse events were observed in this clinical trial: chest discomfort, urinary tract infection, acute heart failure, and amiodarone pneumonia. Chest discomfort was observed on the 37th day after the operation in case 1. After the patient was hospitalized and closely examined for acute coronary syndrome, the event was judged to be a symptom of anxiety. Urinary tract infection was reported in case 2 at 13 weeks postoperatively. The diagnosis was made based on bacteriological examination findings. Case 2 had acute heart failure 40 days after the operation. Because the temperature increased quickly in that season, it was speculated to be caused by the afterload mismatch on the left ventricle owing to the hypertension surge. Amiodarone pneumonia was diagnosed using CT at another hospital 13 weeks after surgery in case 3. Image indicating pneumonia and symptoms improved after discontinuing amiodarone. Causal relationships of all four severe adverse events with iPSC-CMs were excluded (Table 2).

**Table 2:** Severe adverse events.

	Severe adverse events
Case 1	Chest discomfort
Case 2	Urinary tract infection Acute heart failure
Case 3	Amiodarone pneumonia

### 3.4. No evidence of tumor formation

Whole-body FDG-PET was performed 6 months after transplantation to identify tumors that could develop from the transplanted iPSCs. Likewise, case 1, which has already been reported (6), cases 2 and 3 did not show any significant uptake of FDG in FDG-PET scan, eliminating the possibility of tumors (Supplementary Figure S2). In addition, We measured blood levels of four biomarkers of alpha-fetoprotein (AFP), carbohydrate antigen 19-9 (CA19-9), carcinoembryonic antigen (CEA), and human chorionic gonadotropin (hCG) before surgery, 3 months, 6 months, and 1 year after transplantation to detect tumors arising from iPS cells based on a previous report (8). The results showed that hCG was below the sensitivity of the measurement, and the other parameters were also below the reference values at all time points (Supplementary Figure S3).

### 3.5. Serial changes in postoperative cardiac function

**Figure 1:**
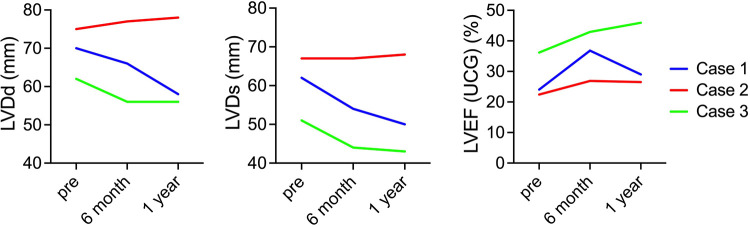
Serial changes in cardiac function. The images show serial changes in LVDd, LVDs, and LVEF on echocardiography. The red, blue, and green charts indicate changes in the first, second, and third cases. LVDd, left ventricular end-diastolic diameter; LVDs, left ventricular end-systolic diameter; LVEF, left ventricular ejection fraction.

**Figure 2:**
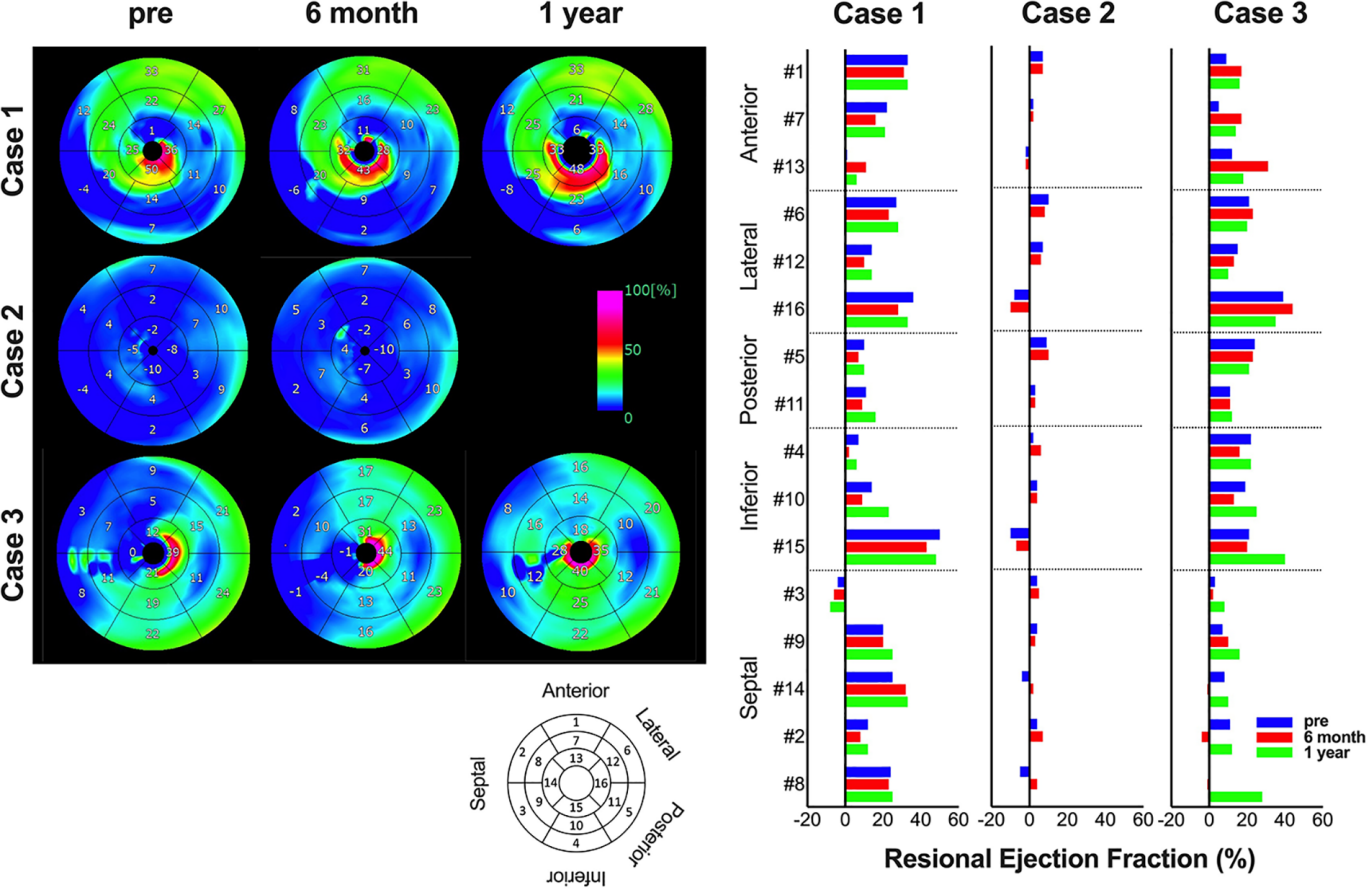
Serial changes in regional cardiac function. The images show serial changes in regional ejection fraction on cardiac computed tomography. Using a model that divides the left ventricle into 17 segments, #1, 7, and 13 are anterior wall, #6, 12, and 16 are lateral wall, #5 and 11 are posterior wall, #4, 10, and 15 are inferior wall, and #3, 9, 14, 2, and 8 are septal wall. The image data are shown on the left, and the graph of quantified values on the right.

Regarding changes in postoperative cardiac function, in cases 1 and 3, the left ventricle, which showed enlargement preoperatively, was reduced 6 months and 1 year after surgery. In addition, the LVEF improved 6 months and 1 year after surgery in cases 1 and 3. In contrast, in case 2, preoperative LVDd/LVDs were enlarged before surgery and did not show noticeable change. Similarly, in case 2, LVEF showed no clear improvement (Figure 1). A similar trend was confirmed using electrocardiogram-gated cardiac CT (Figure 2). In addition, as reported in case 1, the anterolateral wall (segments #1, 7, 13, and 6) of the left ventricle implanted with iPSC-CM sheets showed greater improvement in contraction in case 3; however, there were no apparent changes in ventricular contractility in case 2 (Figure 2).

### 3.6. Serial changes in postoperative myocardial blood flow

Serial changes in myocardial blood flow were measured at rest and under stress using NH_3_-PET, and this ratio was defined as the coronary flow reserve (CFR). Supplementary Figure S4 shows the regional values of 17 segmentations. In case 1, as previously reported, the preoperative value was not obtained. However, a marked increase in blood flow during stress was observed from 1.67 ml/min/g 6 months after surgery to 3.12 ml/min/g 1 year after surgery. Accordingly, the CFR in case 1 increased. In case 2, resting myocardial blood flow was 0.51 ml/min/g preoperatively, 0.57 ml/min/g 6 months after surgery, and 0.47 ml/min/g 1 year after surgery, showing no significant changes. CFR decreased from 2.53 before surgery to 1.11 6 months after surgery and 1.38 1 year after. In case 3, the resting myocardial blood flow decreased from 1.01 ml/min/g preoperatively to 0.69 ml/min/g 6 months after surgery and 0.67 ml/min/g 1 year after surgery. However, stress myocardial blood flow increased from 2.33 ml/min/g preoperatively to 2.54 ml/min/g 6 months after surgery and decreased to 1.63 ml/min/g 1 year after surgery. CFR increased from 2.36 before surgery to 3.76 6 months after surgery and decreased to 2.42 1 year after surgery, which was higher than the values before surgery.

### 3.7. Serial changes in heart failure symptom

**Figure 3:**
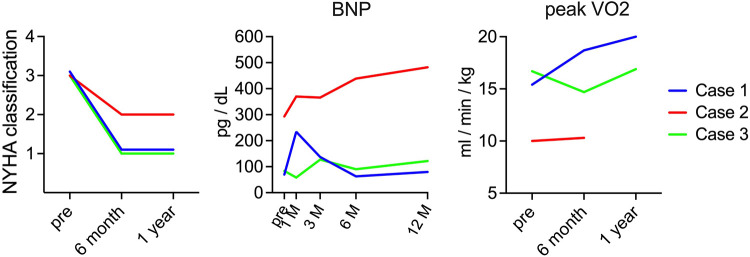
Serial changes in heart failure symptom. The images show serial changes in NYHA classification, BNP, and peak VO_2_. The red, blue, and green charts indicate changes in cases 1, 2, and 3, respectively. NYHA, New York Heart Association; BNP; brain natriuretic peptide.

When the transition of heart failure symptoms was evaluated according to the NYHA classification, all patients had NYHA class 3 heart failure symptoms preoperatively; however, cases 1 and 3 improved to NYHA class I 6 months after surgery, and case 2 improved to NYHA class II 6 months after surgery. In contrast, regarding changes in BNP, cases 1 and 3 showed no significant difference at approximately 100 pg/dl after surgery. In case 2, the preoperative level was 293 pg/dl, which gradually increased after surgery, reaching 482 pg/dl 1 year after the operation. In addition, peak VO_2_, evaluated by cardiopulmonary exercise testing, increased from 15.4 ml/min/kg before surgery to 18.7 ml/min/kg 6 months after surgery and 20 ml/min/kg 1 year after surgery in case 1 and decreased from 16.7 ml/min/kg before surgery to 14.7 ml/min/kg 6 months after surgery but increased to 16.9 ml/min/kg 1 year after surgery in case 3. In contrast, in case 2, the value was as low as 10 ml/min/kg before surgery, and it did not increase at 6 months after surgery, and the surgery could not be performed after 1 year (Figure 3).

### 3.8. HLA typing

The HLA types of iPSC donors and each patient are shown in Table 3. The iPSC donor was homozygous for the six alleles of HLA-A, -B, -C, -DRB1, -DQB1, and -DPB1. Comparing the HLA types of the iPSC donor and each patient clarified the matching of genotype A*24:02 in cases 1 and 3, the matching of HLA-DRB1 serotype DR15 in case 2, and the matching of HLA-DQB1 serotype DQ6 in cases 1 and 2 (Table 3).

**Table 3:** HLA typing; allele (serotype).

	iPS donor	Case 1	Case 2	Case 3
HLA-A	**24:02 (A24)**	**24:02:01 (A24)**	02:01:01 (A2)	**24:02:01 (A24)**
		33:03:01 (A33)	26:05 (A26)	02:06:01 (A2)
HLA-B	**52:01 (B52)**	15:01:01 (B62)	38:02:01 (B38)	51:01:01 (B51)
		44:03:01 (B44)	48:01:01 (B48)	54:01:01 (B54)
HLA-C	**12:02 (Cw12)**	01:02:01 (Cw1)	07:02:01 (Cw7)	01:02:01 (Cw1)
		14:03:01 (Cw14)	08:03:01 (Cw8)	14:02:01 (Cw14)
HLA-DRB1	**15:02 (DR15)**	09:01:02 (DR9)	04:05:01 (DR4)	04:05:01 (DR4)
		13:02:01 (DR13)	15:01:01 (**DR15**)	
HLA-DQB1	**06:01 (DQ6)**	03:03:02 (DQ9)	04:01:01 (DQ4)	04:01:01 (DQ4)
		06:04:01 (**DQ6**)	06:02:01 (**DQ6**)	
HLA-DPB1	**09:01 (DP9)**	04:01:01 (DP4)	02:01:02 (DP2)	02:01:02 (DP2)
		05:01:01 (DP5)		05:01:01 (DP5)

Bold text indicates that it matches the donor's HLA typing.

### 3.9. Serial changes in postoperative immune response

**Figure 4:**
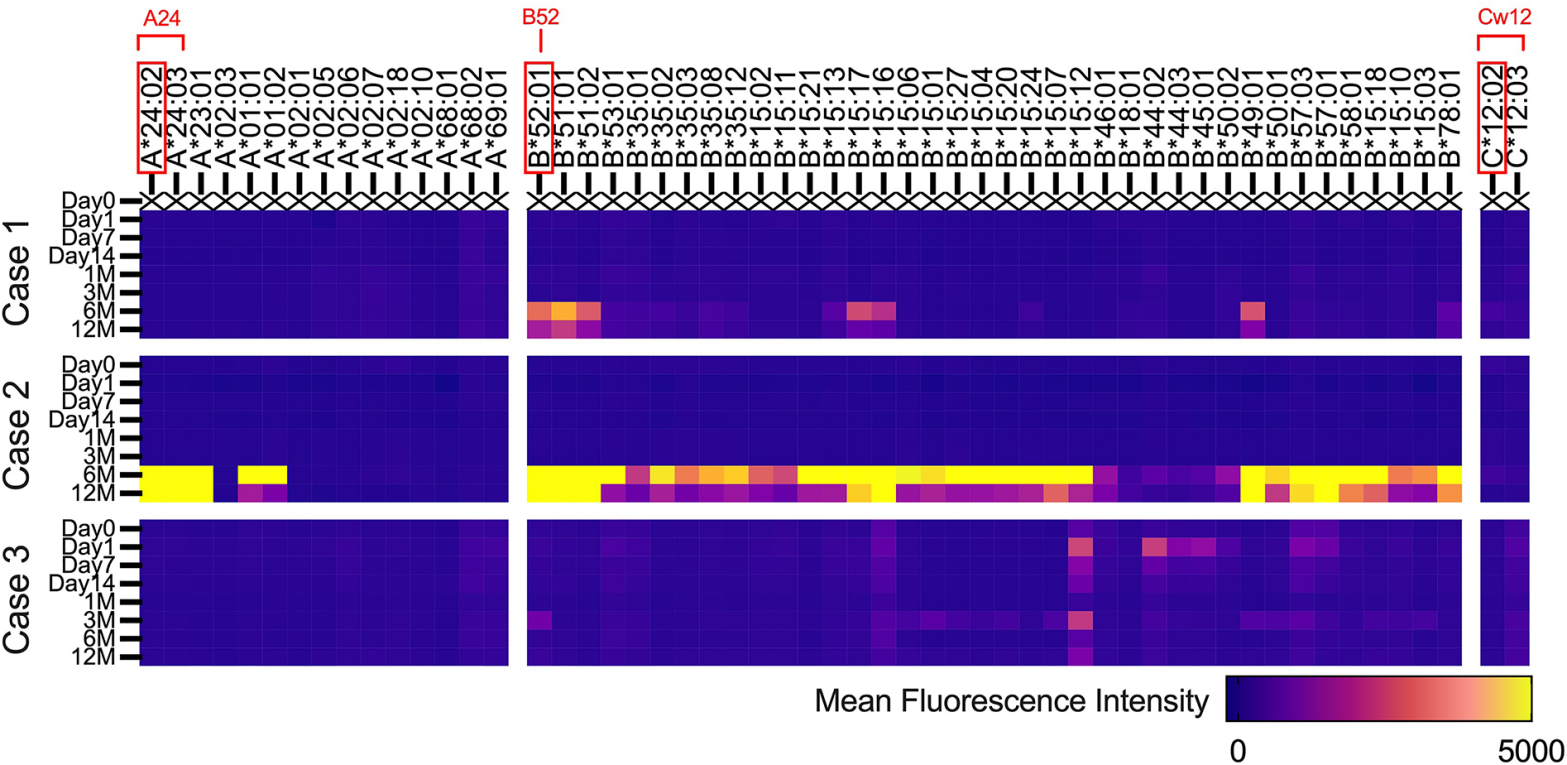
Serial changes in anti-HLA class 1 antibodies. Heatmap showing changes in fluorescence intensities for representative HLA class 1 (HLA-A, B, and C) alleles that are specific or cross-reactive with the HLA type of transplanted iPS cells.

**Figure 5:**
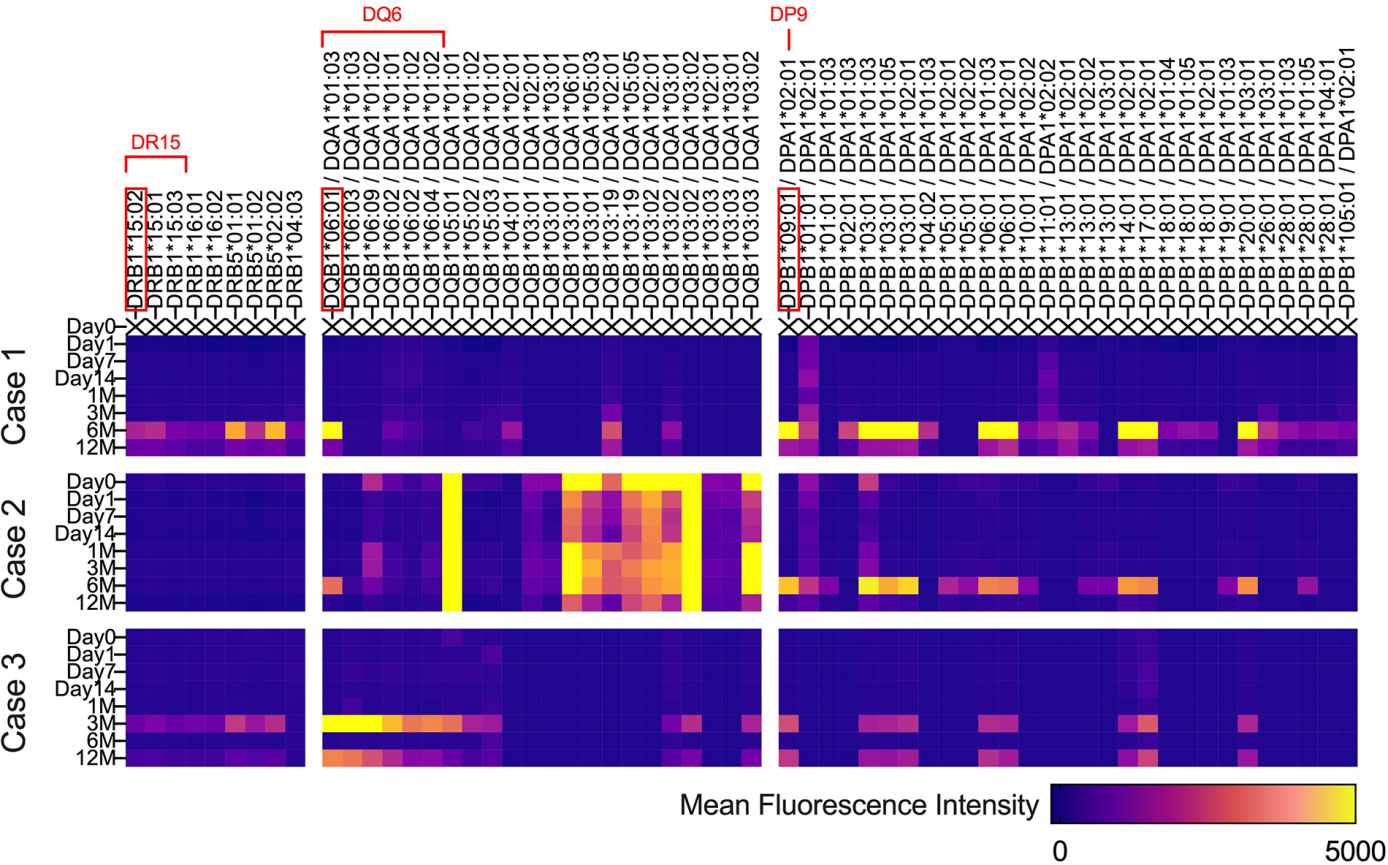
Serial changes in anti-HLA class 2 antibodies. Heatmap showing changes in fluorescence intensities for all evaluated HLA class 2 (HLA-DRB1, DQB1, and DPB1) alleles.

Figure 4 describes the changes in fluorescence intensities for representative HLA class 1 allele (HLA-A, B, and C) that are specific or cross-reactive with the HLA type of transplanted cells. Similarly, Figure 5 shows the changes in fluorescence intensities for all evaluated HLA class 2 alleles (HLA-DRB1, DQB1, and DPB1).

Regarding both HLA class 1 and class 2, all three cases showed increases in fluorescence intensities for multiple anti-HLA antibodies 6 months after transplantation, which was 3 months after the completion of immunosuppressive therapy (Figures 4, 5). In contrast, in case 2, an increase in fluorescence intensity for HLA-DQ was already observed before transplantation (Figure 5).

### 3.10. Evaluation by an external review committee

As described in the Methods section, external evaluation committee members determined the indication for inclusion in the clinical trial based on the evaluation of the preoperative condition of each case. Adverse events and treatment efficacy were evaluated after a 1-year observation period after cell transplantation to determine whether the trial could be continued.

As a result, adverse events during the 1-year postoperative observation period were ruled out as related to the investigational product; therefore, all three patients were judged eligible for inclusion in the trial based on evaluating their preoperative conditions, allowing the trial to continue.

## 4. Discussion

In this first-in-human clinical trial of iPSC-CM transplantation, the first three cases of transplantation and a 1-year follow-up period after transplantation were completed. All adverse events observed during the observation period negatively correlated with iPSC-CM transplantation. In addition, whole-body FDG-PET performed in the latter half of the surgery did not show any tumors that could have been generated from iPSCs (Supplementary Figure S2), thus proving the treatment safe. Improvement in heart failure symptoms was observed in all three cases, especially in two cases (cases 1 and 3). Reduction in left ventricular diameter (Figures 1, 2) and improvement in exercise tolerance (Figure 3) were observed after cell transplantation, suggesting the possibility of a response to cell transplantation treatment.

In this clinical trial protocol, no immunosuppressive agents were administered 3 months after transplantation of allogeneic iPSC-CMs without matching HLA typing, and the transplanted cells were expected to have been lost because of rejection after 3 months. Therefore, the primary mechanism of improvement in cardiac function is the improvement in myocardial blood flow by angiogenesis and functional improvement of residual myocytes, as previously observed in preclinical studies (3, 4). Myocardial blood flow and CFR evaluated using NH_3_-PET in this study were similar or improved after 6 months in cases 1 and 3 compared with those before transplantation, whereas they were decreased in case 2. In addition, myocardial blood flow, CFR, left ventricular diameter, and contractility improved in cases 1 and 3 at 6 months after transplantation, while there was no clear improvement in left ventricular function in case 2. Therefore, it is suggested that in cases 1 and 3, angiogenesis from the transplanted cells occurred during the immunosuppression period of 3 months after transplantation, and the functional recovery of the remaining myocardium obtained from the improved blood flow was maintained even 6 months after the loss of transplanted cells. In contrast, in case 2, the transplanted cells did not have a sufficient angiogenic effect, which could have resulted in the lack of improvement in cardiac function. Therefore, immune response to the transplanted cells during the immunosuppression period of 3 months after cell transplantation, as conducted in this clinical trial protocol, may be essential to obtain the therapeutic effect.

Because the histological evaluation of the transplanted tissue was practically impossible, we searched for the presence of anti-HLA antibodies that are specific or cross-reactive with the transplanted iPSCs, as generally performed in allogeneic heart transplantation, to evaluate immune response (9). As shown in Figures 4, 5, 3 months after surgery, during which each patient continued to take immunosuppressants, new emergence of anti-HLA antibodies that could react with transplanted iPSCs was not evident in all cases, except for anti-HLA antibodies against HLA-DQ in case 2. Therefore, we speculate that immune rejection against the transplanted tissue was suppressed in cases 1 and 3, whose cardiac function improved. In contrast, in case 2, whose cardiac function did not improve, anti-HLA antibodies against HLA-DQ presented before surgery may have caused the immune rejection. However, at present, it is not possible to demonstrate a causal relationship between the suppression of immune rejection by transplanted iPSCs and the improvement in cardiac function derived from transplanted iPSCs. Therefore, it is crucial to verify the effect of iPSC-CM on cardiac function from the viewpoint of immune response in the future.

Furthermore, residual myocardium, generally referred to as hibernating myocardium (10), is functionally impaired owing to ischemia but can recover through improvement in blood flow and can improve myocardial blood flow by iPSC-CM transplantation. We previously showed that in autologous skeletal myoblast sheet transplantation, the primary mechanism of cardiac function improvement by similar angiogenic therapy is considered to be the improvement in cardiac function, and the lack of progression of left ventricular enlargement is a factor that predicts responders who can obtain therapeutic effects (11). These results suggest that there is not much residual myocardium in cases with progressive left ventricular enlargement (12), which may also be used to predict responders who will benefit from treatment in this clinical trial. In case 2 of this trial, the preoperative LVESV was 305 ml, which was markedly increased compared to that in case 1 (182 ml) and case 3 (122 ml), and even autologous skeletal myoblast sheet transplantation could not have been a responder (11). Therefore, in addition to the immune response to the transplanted cells, it is also possible that the small amount of residual myocardium in case 2 did not respond to angiogenic therapy. It will be important to avoid immune response and predict treatment efficacy by quantifying the residual myocardium to generalize this treatment method in the future.

This study had a limitation. This is the first clinical trial using iPSC-CMs with the main objective of confirming safety. Therefore, the number of cases is overwhelmingly limited to conclude therapeutic efficacy and therapeutic mechanism. After ensuring safety in this clinical trial, increasing the number of cases and conducting a study to assess the therapeutic effect is necessary.

In conclusion, allogeneic iPSC-CM transplantation can be performed safely, supporting further clinical trials. The effect of preoperative HLA antibodies on the therapeutic effect should be investigated in more cases in the future.

## Data Availability

The original contributions presented in the study are included in the article/**Supplementary Material**, further inquiries can be directed to the corresponding author.
