# Supplementary material for: Safety confirmation of induced pluripotent stem cell-derived cardiomyocyte patch transplantation for ischemic cardiomyopathy: first three case reports
Source: Front Cardiovasc Med. 2023 Sep 15;10:1182209. doi: 10.3389/fcvm.2023.1182209 (PMC10540447; doi:10.3389/fcvm.2023.1182209)
Supplement: Supplementary file 1 [file Table1.docx]

# Supplementary Figure Legends

**Supplementary Figure 1. Transplantation of hiPSC-CM patches onto the heart surface**

HiPSC-CM patch was transplanted into the epicardium of the anterior and lateral walls of the LV. The picture shows the Case 3.

**Supplementary Figure 2. FDG-PET imaging at 6 months after transplantation**

FDG-PET in cases 2 and 3 showed no apparent abnormal accumulation in the whole body and heart.

**Supplementary Figure 3. Serial changes in tumor biomarkers**

The graphs show serial changes in blood levels of alpha-fetoprotein (AFP), carbohydrate antigen 19-9 (CA19-9), and carcinoembryonic antigen (CEA) before surgery, 3 months, 6 months, and 1 year after transplantation.

**Supplementary Figure 4. Serial changes in MBF and CFR for the 17-segment model**

The graphs show serial changes in MBF and CFR on NH_3_-PET for the 17-segment model.
