# Supplementary figures and images for: Safety confirmation of induced pluripotent stem cell-derived cardiomyocyte patch transplantation for ischemic cardiomyopathy: first three case reports
Source: Front Cardiovasc Med. 2023 Sep 15;10:1182209. doi: 10.3389/fcvm.2023.1182209 (PMC10540447; doi:10.3389/fcvm.2023.1182209)

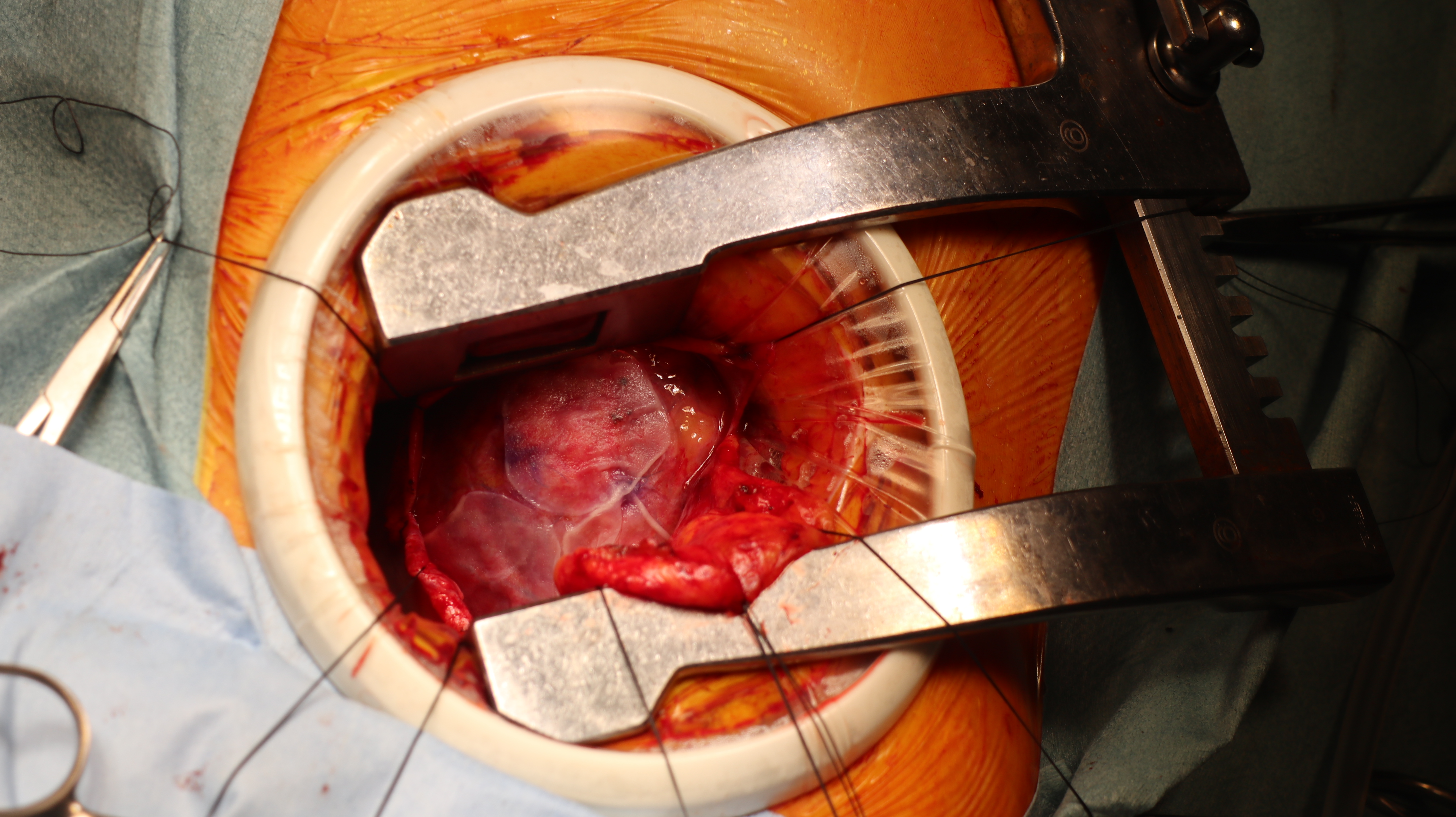

Supplement: Supplementary file 2 [file Image1.jpeg]

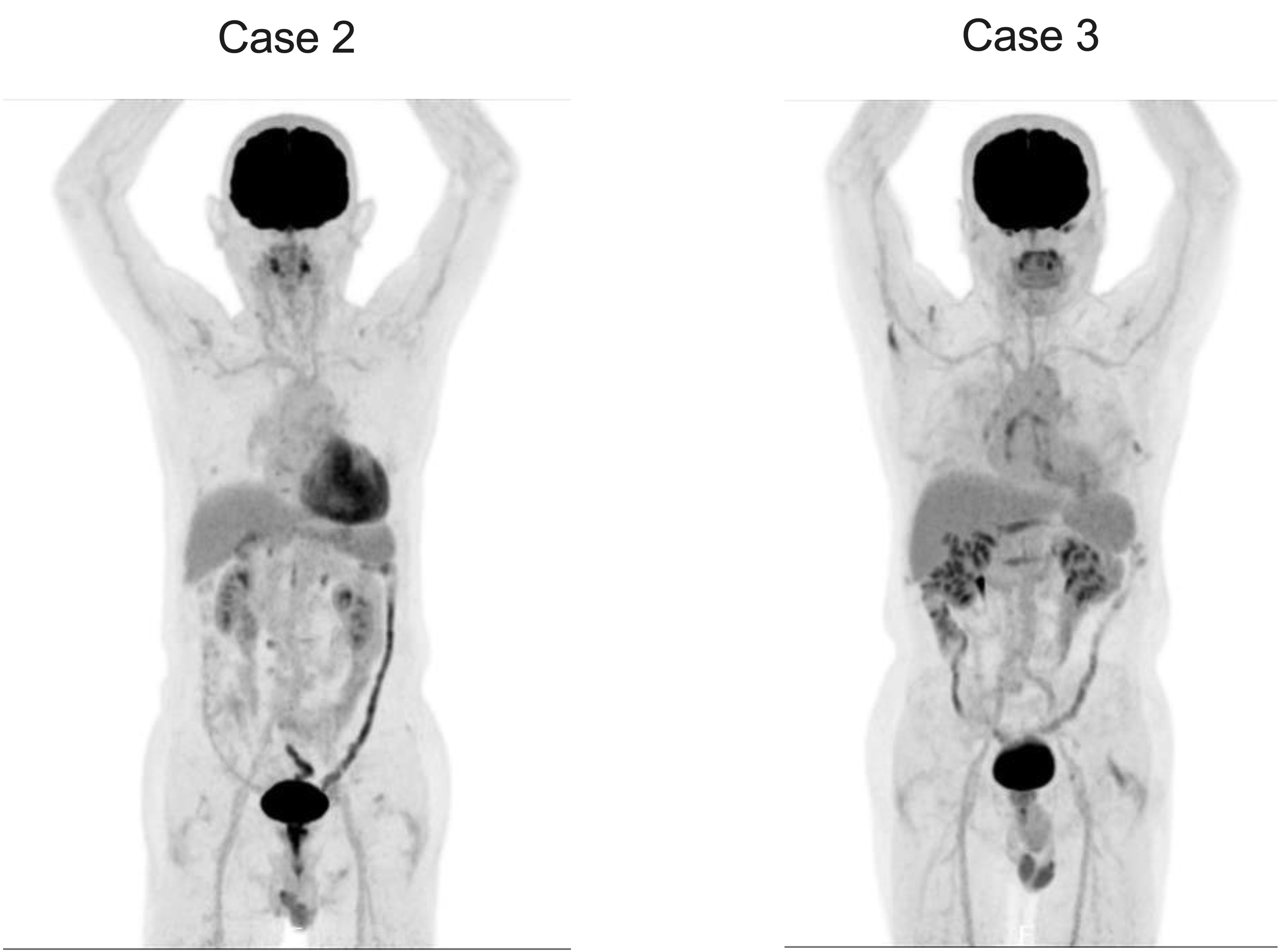

Supplement: Supplementary file 3 [file Image2.jpeg]

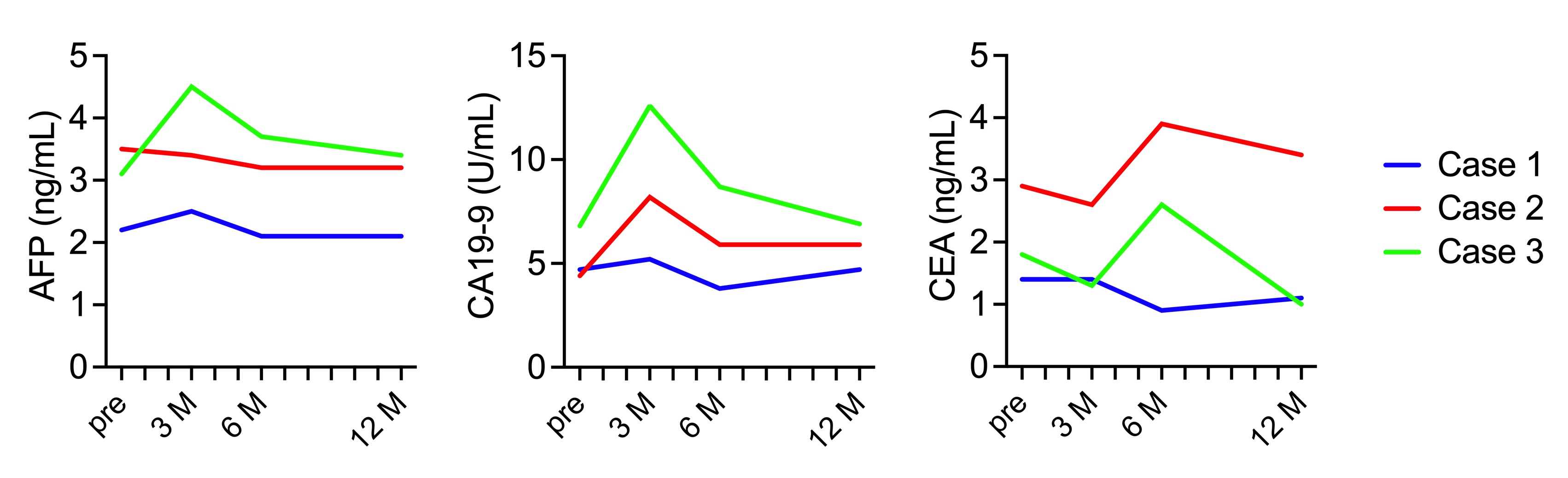

Supplement: Supplementary file 4 [file Image3.jpeg]

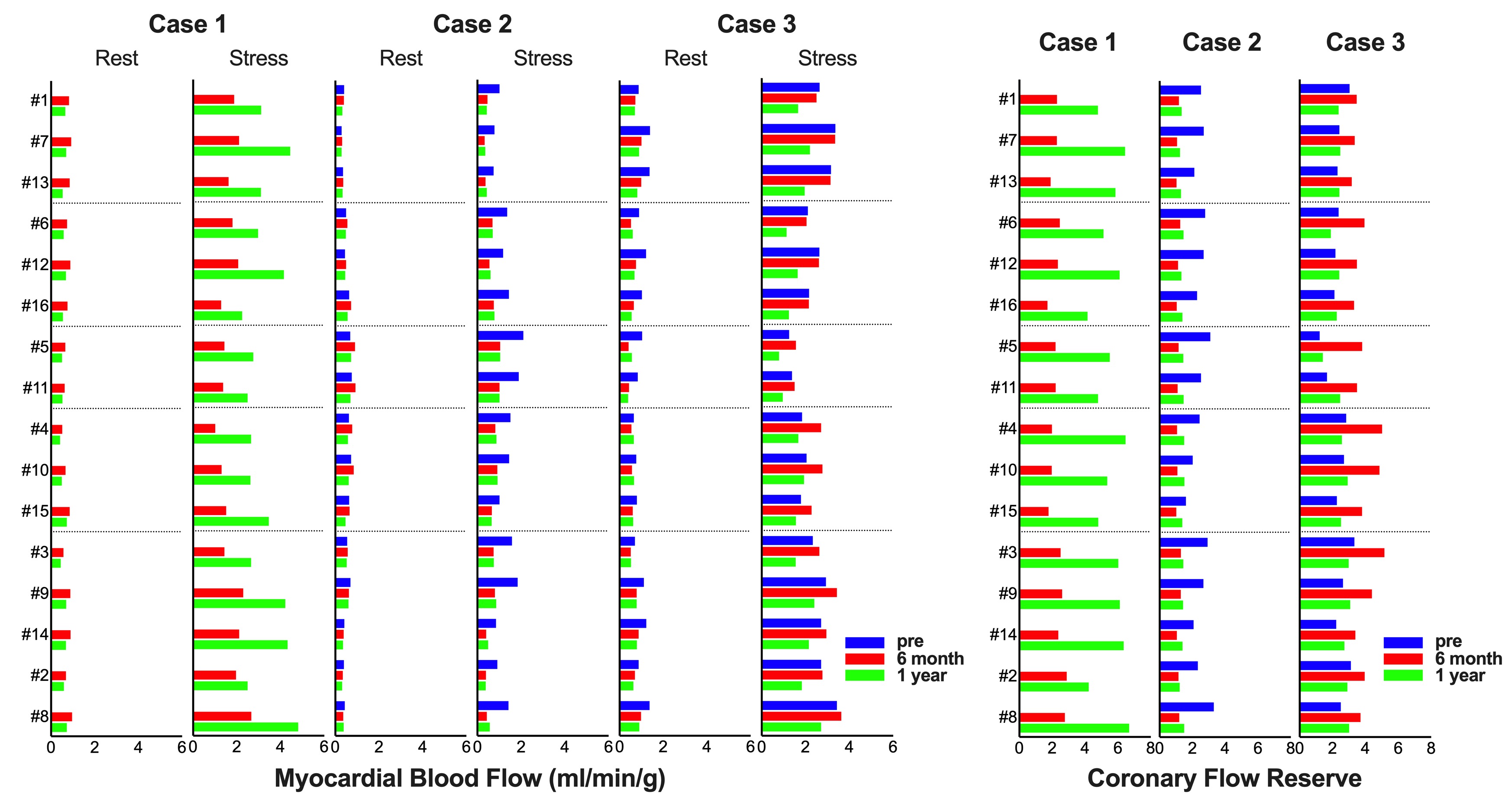

Supplement: Supplementary file 5 [file Image4.jpeg]
